# BRCA1-Dependent Transcriptional Regulation: Implication in Tissue-Specific Tumor Suppression

**DOI:** 10.3390/cancers10120513

**Published:** 2018-12-14

**Authors:** Xiaowen Zhang, Rong Li

**Affiliations:** Department of Biochemistry & Molecular Medicine, School of Medicine & Health Sciences, The George Washington University, Washington, DC 20037, USA

**Keywords:** BRCA1, transcriptional regulation, epigenetic regulation, chromatin organization

## Abstract

Germ-line mutations in breast cancer susceptibility gene 1 (*BRCA1*) predominantly predispose women to breast and ovarian cancers. BRCA1 is best known for its functions in maintenance of genomic integrity including repairing DNA double-strand breaks through homologous recombination and suppressing DNA replication stress. However, whether these universally important BRCA1 functions in maintenance of genomic stability are sufficient to account for its tissue-specific tumor-suppressing function remains unclear. Accumulating evidence indicates that there are previously underappreciated roles of BRCA1 in transcriptional regulation and chromatin remodeling. In this review, we discuss the functional significance of interactions between BRCA1 and various transcription factors, its role in epigenetic regulation and chromatin dynamics, and BRCA1-dependent crosstalk between the machineries of transcription and genome integrity. Furthermore, we propose a model of how transcriptional regulation could contribute to tissue-dependent tumor-suppressing function of BRCA1.

## 1. Introduction

Approximately 0.2% to 0.3% of the general population in the United States carries germ-line mutations in the tumor suppressor gene *BRCA1* (*BRCA1^mut/+^*) [1,2]. Unlike tumor suppressors such as p53 that are implicated in a broad spectrum of cancers, BRCA1 functions in a gender- and tissue-specific manner. *BRCA1* mutation-carrying women have significantly higher risk of developing breast and ovarian cancers compared to the general population, with an estimated cumulative risk of 65% and 39% by the age of 70, respectively [3,4,5]. By comparison, *BRCA1* mutation-carrying men have an estimated cumulative risk of 1.2% of developing breast carcinoma at the same age [6]. *BRCA1*-mutated breast cancers are typically more aggressive and higher grade with an increased rate of *TP53* mutations [7,8,9,10]. In addition, these *BRCA1*-associated breast tumors tend to be triple-negative for estrogen receptor α (ER-), progesterone receptor (PR-), and HER2 (HER2-), making it more challenging to develop targeted therapies [11,12,13,14]. PARP inhibitor olaparib has recently been approved by the US Food and Drug Administration (FDA) to treat *BRCA*-mutated metastatic breast cancer; and several other PARP inhibitors are currently under clinical development [15,16]. Despite these exciting developments, chemotherapy is still the first-line therapy for *BRCA1*-related breast cancers [17,18]. 

**Figure 1:**
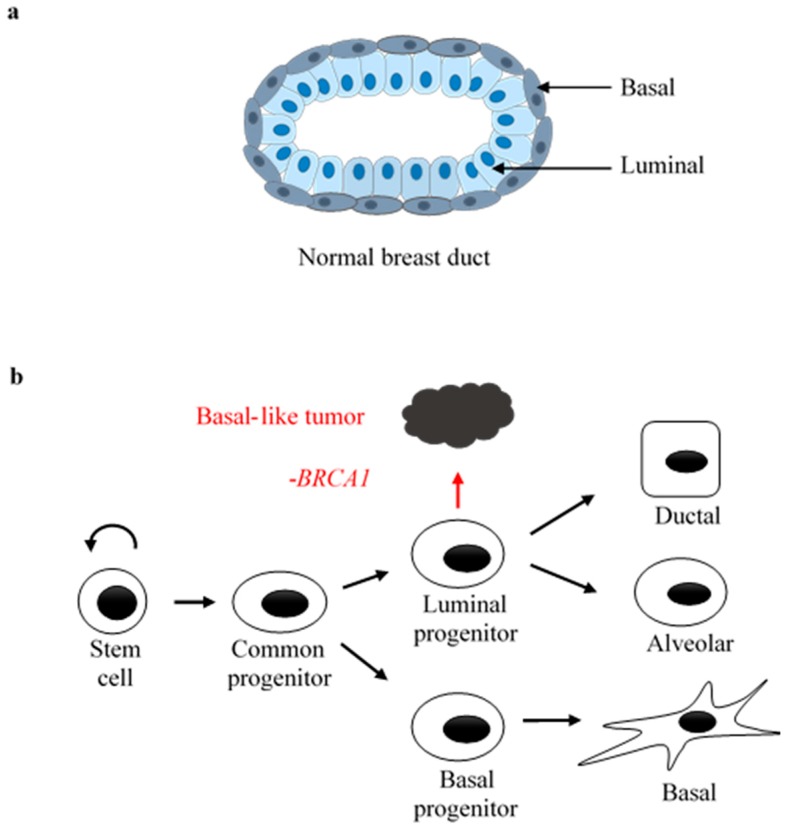
The developmental hierarchy of human breast. (**a**) Cross-section of a normal breast duct. (**b**) Breast epithelial hierarchy and *BRCA1*-associated breast cancer.

Breast epithelia consist of two layers of epithelial cells (Figure 1a): the inner layer with luminal progenitors and ductal/alveolar cells, and the outer layer with mammary stem cells and myoepithelial cells [19]. The luminal and basal cell lineages express distinct sets of fate-determining genes that fulfill lineage-specific functions. For example, mature luminal cells express ERα and PR, which, together with a number of additional luminal lineage-specific transcription factors, regulate side branching and alveologenesis in the breast epithelia [20,21]. *BRCA1* mutation leads to aberrant luminal lineage development. Of note, luminal progenitor cells from disease-free *BRCA1* mutation carriers (*BRCA1^mut/+^*) exhibit deficiency to differentiate into mature luminal cells [22,23]. Besides, luminal differentiation-associated gene expression is significantly reduced in *BRCA1^mut/+^* breast epithelium versus their non-carrier controls [22,23]. Furthermore, in vitro proliferation of *BRCA1^mut/+^* luminal progenitors is less growth factor-dependent than their *BRCA1^+/+^* counterparts [22], consistent with the notion that these mutant progenitors are aberrantly proliferative yet defective in differentiation. The deficiencies observed in *BRCA1* mutated clinical samples were corroborated by work using genetically engineered mouse models with lineage-specific deletion of mouse *Brca1* [22,24]. More recent studies indicate that the RANK-RANKL axis, a key player that mediates paracrine actions in luminal homeostasis, is abnormally activated in breast epithelia of *BRCA1* mutation carriers [25]. Ostensibly normal *BRCA1^mut/+^* breast tissue has a higher percentage of RANK^+^ luminal progenitors, cells highly proliferative and prone to DNA damage [25]. Inhibition of RANKL, the ligand of RANK, attenuates mammary tumorigenesis in *Brca1*-deficient mice [25]. Thus, despite the fact that *BRCA1*-associated breast tumors tend to be basal-like and triple-negative, both clinical and preclinical studies strongly suggest that luminal progenitor cells are the cell-of-origin of *BRCA1*-mutated breast cancers [22,23,26] (Figure 1b). While it is abundantly clear that germ-line *BRCA1* mutations confer tissue- and cell lineage-specific cancer, the mechanism underlying the context-dependent dysfunction of cancer-predisposing *BRCA1* mutations remains largely unknown.

BRCA1 is best known for maintenance of genomic integrity through its functions in homologous recombination (HR)-dependent repair of double-strand DNA breaks [27,28,29], regulation of cell cycle checkpoints [30,31], and suppression of DNA replication stress [32]. While these BRCA1-dependent processes most likely contribute to its tumor suppressor function, they may not be sufficient to explain the aforementioned longstanding conundrum in *BRCA1*-related cancer biology, namely, the sex/tissue selectivity and luminal-to-basal lineage conversion during tumorigenesis. Besides its well-documented functions in maintenance of genome stability, BRCA1 is also implicated in transcriptional regulation and chromatin reorganization [30,33,34,35,36,37,38], processes that primarily dictate normal tissue development and lineage-specific cell differentiation. Here, we summarize recent findings concerning the roles of BRCA1 in transcriptional regulation and discuss their potential contributions to the tissue- and lineage-specific tumor suppressor functions of BRCA1.

## 2. Functional Interaction between BRCA1 and Transcription Factors

**Figure 2:**
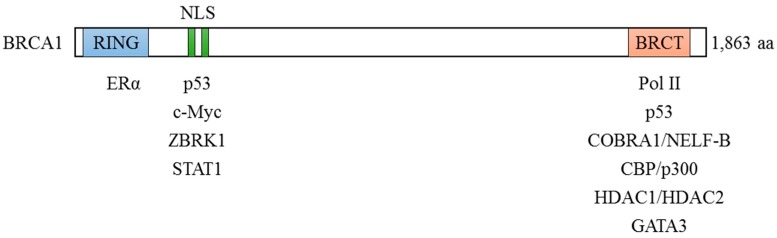
Interactions between BRCA1 and transcription factors.

A potential role of BRCA1 in transcriptional regulation was first described two decades ago [39]. When fused to a heterologous DNA binding domain, the carboxyl-terminus of BRCA1 was shown to activate transcription in both budding yeast and mammalian cells [39,40,41]. Interestingly, cancer-predisposing *BRCA1* mutations abolished BRCA1-mediated transcriptional activation, suggesting a possible role of transcriptional regulation in mediating tumor suppressing function of BRCA1 [39]. It was later found that BRCA1 was co-purified with the RNA polymerase II (Pol II) holoenzyme complex [35]. This interaction was through a direction interaction between the C-terminus of BRCA1 and RNA helicase A, a component of the Pol II holoenzyme [35,42]. In addition to its interaction with basal transcription machinery, BRCA1 has also been shown to bind to several known transcription factors, including p53 [37,43], estrogen receptor alpha (ERα) [44], cofactor of BRCA1 (COBRA1) [34], c-Myc [45], ZBRK1 [46], GATA3 [47] and STAT1 [48] (Figure 2). Excellent reviews on this topic can be found elsewhere [30,49,50]. In this review, we discuss the functional significance of the interactions between BRCA1 and some of these transcription factors.

### 2.1. BRCA1 with p53

Two groups independently discovered the interaction between BRCA1 and p53 [37,43]. BRCA1 was shown to physically interact with p53 in vitro and in vivo and stimulate p53-dependent gene expression [37,43]. The p53/BRCA1 interaction is mediated by both the amino-terminal domain (aa 224–500) and the second BRCT domain (aa 1760–1863) of BRCA1 [37,51]. Interestingly, the p53 coactivator function of BRCA1 only manifests in activation of growth arrest-, but not apoptosis-related transcriptional targets of p53 [52,53]. Besides assisting p53 as a transcriptional coactivator, BRCA1 was also reported to stabilize p53 protein through transcriptional activation of p14^ARF^, another tumor suppressor [54]. Conversely, p53 has been shown to transcriptionally repress BRCA1 expression, therefore forming a possible feedback loop [55,56]. 

A functional interaction between BRCA1 and p53 was observed from studies of several genetically modified mouse models. Homozygous *Brca1* null leads to embryonic lethality [57,58,59,60]. However, survival of *Brca1^Δ5-6/Δ5-6^* and *Brca1^Δ2/Δ2^* embryos are prolonged by homozygous *Trp53* deletion [57,58,60]. In a different *Brca1* mouse model, elimination of one *Trp53* allele (*Trp53^+/−^*) is sufficient to completely rescue *Brca1^Δ11/Δ11^* embryonic lethality [59]. The p53-associated rescue is most likely due to the loss of p53-dependent apoptosis and G1/S checkpoint, allowing *Brca1*-null cells to proliferate in the presence of DNA damage [59]. Interestingly, *Brca1^Δ11/Δ11^; Trp53^+/−^* mice, although able to survive to adulthood, exhibit premature aging phenotype [61]. Mouse mammary luminal epithelium-specific knockout of *Brca1* (*Wap-cre; Brca1^11f/11f^* and *MMTV-cre; Brca1^11f/11f^*) results in mammary gland developmental defect and an increased rate of apoptosis [62]. Tissue-specific *BRCA1* knockout mice develop spontaneous mammary tumors at a long latency, and the tumor formation is significantly accelerated with *Trp53* inactivation [62,63]. Importantly, most *Brca1* knockout tumors have spontaneous *Trp53* mutation, suggesting that loss of p53 is required for tumorigenesis [62]. This is consistent with the aforementioned phenomenon that *BRCA1*-associated human breast tumors have significantly increased chance of carrying *TP53* mutations, compared to *BRCA1*-unrelated breast tumors [7,64]. Despite the genetic interactions between BRCA1 and p53 during normal tissue development and tumorigenesis, it remains unclear whether these functional interplays are dependent on their physical interaction.

### 2.2. BRCA1 with ERα

BRCA1 has an intertwined relationship with ERα signaling. BRCA1 stimulates transcription of *ESR1*, the gene that encodes ERα [65]. This transactivation ability of BRCA1 is mediated by OCT1, a site-specific transcription factor that binds to the *ESR1* promoter and recruits BRCA1 through the OCT1/BRCA1 interaction [65]. On the other hand, BRCA1 inhibits both ligand-dependent and ligand-independent transcriptional activity of ERα [66,67]. Notably, tumor-associated *BRCA1* mutants are defective in suppressing ERα transcriptional activity [66,67]. The BRCA1-associated suppression of ERα transcriptional activity can be explained by several mechanisms. First, BRCA1 directly interacts with ERα in vitro and in vivo and inhibits its activity [66]. The BRCA1/ERα interacting domains have been mapped to the N-terminal of BRCA1 (aa 1–300) and the C-terminal activation function 2 (AF-2) domain of ERα, respectively [44]. Second, BRCA1 down-regulates p300, a well-known ERα coactivator [68,69]. Indeed, ectopic expression of p300 rescues the BRCA1 inhibition of ERα activity [70]. Third, mono-ubiquitination of ERα by BRCA1 suppresses ERα activity [71]. In support, a BRCA1 mutant that disrupts its ubiquitin ligase activity abolishes the ability of BRCA1 to inhibit ERα [71]. These mechanisms are not mutually exclusive, and a combination of more than one could contribute to the reported BRCA1-mediated repression of the vast majority of estrogen-responsive genes [72]. Adding to this complexity, BRCA1 itself is an estrogen-responsive gene [73,74]. Whether ERα directly binds to BRCA1 promoter is still under debate, but 17-β-estradiol (E2) treatment can stimulate BRCA1 expression in mammary gland of ovariectomized mice [73]. In summary, the current data suggest an interrelated mutual regulation between BRCA1 and ERα signaling.

The physical and functional interactions between BRCA1 and ERα provide a plausible molecular explanation for the preferential association of *BRCA1* mutations with cancer risk in estrogen-responsive tissues/organs. BRCA1 deficiency leads to an expanded luminal progenitor population and deficiency in luminal cell differentiation [22,24]. This could be explained, at least partially, by compromised BRCA1 ability in stimulating ERα expression [65]. In further support, Liu et al. reported that BRCA1 plays an important role in differentiation of ER-negative stem/progenitor cells to ER-positive luminal cells [75]. Using mouse models and/or human breast tissues, it was shown that *BRCA1*-associated basal-like breast tumors originate from luminal progenitor cells [22,23,26]. These findings raise two outstanding questions. First, luminal progenitor cells in the post-pubertal mammary glands are slow replicating, largely ERα-negative cells [19,76,77]. Therefore, the functional interaction between BRCA1 and ERα during tumorigenesis, if any, could work in a paracrine manner instead of being mediated by a direct protein-protein interaction in the same epithelial cell type. In support of this possibility, Nolan et al. identified a subset of luminal progenitor cells that express RANK, an important paracrine mediator of hormonal signaling [25]. RANK^+^ luminal progenitors in *BRCA1* mutation carriers are highly proliferative, and inhibition of its ligand RANKL attenuates mammary tumor formation in *Brca1* knockout mice [25]. Another related question concerns how BRCA1-deficient luminal progenitor cells develop into basal tumors. In this regard, recent work by the Kuperwasser’s group suggests that Slug, a transcription factor involved in mammary development and lineage commitment, is aberrantly expressed in BRCA1-deficient breast tissues [23,78]. 

### 2.3. BRCA1 with COBRA1/NELF-B

Our group first demonstrated that BRCA1 interacts with cofactor of BRCA1 (COBRA1) through its BRCT domain [34]. Interestingly, cancer-predisposing BRCA1 mutants A1708E and M1775R exhibit increased affinity for COBRA1 [34]. In an independent study, COBRA1 was identified as the B subunit of the negative elongation complex (NELF), which pauses Pol II at the promoter-proximal region and attenuates transcription elongation [79]. NELF-mediated Pol II pausing is a crucial regulatory step of transcription in metazoans, lack of which is detrimental to early embryogenesis and tissue homeostasis [24,80,81,82,83]. BRCA1 and COBRA1 are shown to concertedly regulate transcription [24,84]. The functional interaction between these two proteins is best demonstrated in a mammary epithelium-specific knockout mouse model [24,81]. Homozygous *Cobra1* knockout in mouse mammary gland (*MMTV-cre; Cobra1^f/f^*) leads to severe developmental defect accompanied by alveologenic and lactogenic deficiencies [24]. Consistent with its role in Pol II pausing, the gene expression profiles in *Cobra1* knockout mammary glands are significantly different from their wild-type littermates, especially for those genes previously identified as puberty-related [24]. Neither co-deletion of the *Ink4a/Arf* locus or *Trp53* rescues these deficiencies, suggesting that the developmental defects in *Cobra1* knockout mostly likely are not caused by senescence, cell cycle arrest or apoptosis [24]. In stark contrast, all developmental defects associated with loss of COBRA1 are largely rescued by co-deletion of *Brca1* exon 11 (*MMTV-cre; Cobra1^f/f^; Brca1^11f/11f^*) [24]. Concordantly, aberrant pubertal gene expression in *Cobra1* knockout mammary gland is partially restored by co-deletion of *Brca1* exon 11, indicating that BRCA1 antagonizes COBRA1-dependent transcription program in mammary epithelia [24]. Notably, *Brca1* point mutants that abrogate either its E3 ligase activity or the phospho-recognition property fail to rescue the mammary developmental defects in *Cobra1* knockout mice [81]. Therefore, it is reasonable to speculate that BRCA1 exon 11 encodes the region important to antagonize COBRA1-mediated transcriptional regulation. 

Further functional characterization of the above-mentioned mouse genetic models reveals that *Cobra1* deletion reduces *Brca1*-associated mammary tumorigenesis [85], thus clearly demonstrating mutual functional antagonism between these two genes in both normal tissue development and mammary tumor formation. Cell line-based studies showed that BRCA1 is responsible for elimination of R-loops, RNA-DNA hybrids and by-products of transcription [86,87]. Importantly, persistent R-loops are known to threaten genome integrity and change gene expression profiles [88]. Using cancer-free human breast tissues from *BRCA1* mutation carriers and non-carriers, we conducted a genome-wide survey of BRCA1-associated R-loop signals. We found that *BRCA1* mutation-associated R-loop accumulation only occurs in luminal epithelial cells, which is reminiscent of the lineage-specific cell-of-origin for *BRCA1*-associated breast tumors [85]. In addition, these BRCA1 deficiency-associated R-loops preferentially accumulate at transcription start sites with paused Pol II, the transcriptional event controlled by COBRA1/NELF-B [85]. Functional antagonism between BRCA1 and COBRA1 in R-loop regulation can be recapitulated in human breast cancer cells in vitro [85]. Furthermore, genetic ablation of *Cobra1* mitigates R-loop accumulation in *Brca1*-ablated mouse mammary epithelium, suggesting that *Brca1* deletion-associated R-loop accumulation is largely caused by the action of COBRA1 [85]. It is worth noting that neither the double-strand break repair defect nor DNA replication stress associated with BRCA1 deficiency was rescued in *Cobra1/Brca1* double knockout [85]. Together with the finding that co-deletion of the two genes significantly reduces *Brca1*-associated mammary tumorigenesis, these results indicate that attenuation of Pol II pausing-induced R-loops likely contributes to the tumor suppressor function of BRCA1 [85].

## 3. The Roles of BRCA1 in Epigenetic Regulation

Epigenetics, including DNA methylation and histone modifications, is a critical transcriptional regulatory mechanism [89,90]. BRCA1 alters epigenetics through its physical interaction with, and transcriptional regulation of known epigenetic modifiers. In addition, as a ubiquitin E3 ligase, BRCA1 directly ubiquitylates histones. Here we summarize several recent studies that elucidate the roles of BRCA1 in epigenetic control.

### 3.1. BRCA1 in DNA Methylation

DNA methylation, covalent addition of a methyl group to the fifth position of the cytosine ring of DNA, is a stable repressive epigenetic mark that silences transcription [91,92]. It is an evolutionary conserved phenomenon that promoter methylation negatively correlates with gene expression [93]. Global DNA hypomethylation and promoter hypermethylation are common features in most cancer types including breast cancer [94,95,96]. *BRCA1*-associated breast tumors, in particular, exhibit less DNA methylation compared with sporadic breast tumors [97,98,99]. There are two groups of DNA methyltransferases (DNMTs): (1) de novo methyltransferases DNMT3A and DNMT3B that put the initial methyl groups onto DNA, and (2) methylation maintenance enzyme DNMT1 that copies the methylation pattern from the template strand to the newly synthesized strand after DNA replication [100,101,102]. As detailed below, BRCA1 is reported to associate with both groups [99,103]. 

BRCA1 physically interacts with de novo methyltransferase DNMT3B and modulates heterochromatin methylation [103]. This interaction was demonstrated in a *Wip1* deletion model [103]. Wip1 is a p53-induced serine/threonine phosphatase, and its overexpression is observed in various cancers [104,105]. Loss of Wip1 in mouse germ cells and human cancer cells leads to dramatically increased global 5-methylcytosine level, especially at *L1 LINE* retrotransposons [103]. It is worth noting that *L1 LINE* comprises 17% of the human genome [106]. The marked enrichment of DNA methylation at *L1 LINE* is associated with decreased level of *L1 LINE* transcripts [103]. Surprisingly, the increased global level of 5-methylcytosine, elevated DNA methylation at *L1 LINE*, and reduced *L1 LINE* mRNA expression in Wip1-depleted cells are all rescued by either a single allele deletion of ATM or depletion of BRCA1 [103]. This result puts the actions of ATM and BRCA1 between the Wip1 loss and elevated DNA methylation on retroelements. Further investigation confirms previous reports that *Wip1* deletion constitutively activates ATM-dependent DNA damage response, which subsequently turns on the downstream effector BRCA1 [103,107,108]. Activated BRCA1 forms a complex with DNMT3B and heterochromatin protein 1 (HP1) that methylate *L1 LINE* sequences [103,109]. Importantly, the involvement of BRCA1 in facilitating DNA methylation is ATM-dependent, since mutation of the ATM phosphorylation sites on BRCA1 significantly attenuates the BRCA1-DNMT3B-HP1 complex assembly [103]. In further support, overexpression of Wip1 decreases DNA methylation of *L1 LINE,* accompanied by significantly increased *L1 LINE* mRNA level. Unmethylated DNA serves as substrate of cytidine deaminases [110]. If not properly repaired, cytidine deamination generates C-to-T mutations [110]. Indeed, the copy number of *PPM1D*, the gene that encodes Wip1, positively correlates with C-to-T mutation load in primary human breast tumors [103]. Thus, a potential role of ATM/BRCA1 signaling in regulating global DNA methylation could contribute to genome integrity. 

In addition to its physical interaction with de novo methyltransferase DNMT3B, BRCA1 also regulates transcription of methylation maintenance enzyme DNMT1 and prevents global DNA hypomethylation [99]. BRCA1 is associated with a putative OCT1-binding motif on the DNMT1 promoter in both human and mouse cells, and its binding leads to a transcriptionally active configuration of the promoter [99]. *Brca1^Δ11/Δ11^* mice exhibit dramatically decreased level of DNMT1, which causes global DNA hypomethylation, loss of genomic imprinting, and an open chromatin configuration globally. Importantly, BRCA1 deficiency in mouse mammary gland leads to marked reduction of promoter methylation and mRNA overexpression of several proto-oncogenes including c-Myc, Ha-Ras, and c-Fos [99]. In primary human breast tumors, there is a positive correlation between protein levels of BRCA1 and DNMT1 [99]. Furthermore, *BRCA1*-mutated breast cancer is associated with reduced *DNMT1* transcription when compared with non-mutated breast cancer [111]. The transcriptional link between BRCA1 and DNMT1 strongly indicates a function of BRCA1 in global DNA methylation, thus providing another plausible mechanism for *BRCA1* mutation-associated DNA hypomethylation and breast cancer formation.

Although BRCA1 positively regulates DNA methylation at a global level, it suppresses gene-specific promoter methylation through its interaction with EZH2 [112,113]. EZH2, a subunit of the Polycomb repressive complex 2 (PRC2), interacts with DNMTs and directly controls DNA methylation [114]. BRCA1 functions as an inhibitor for EZH2 recruitment and activity [115]. In breast cancer cell lines, BRCA1 is shown to positively regulate *FOXA1* and *FOXO3* expression by interfering with EZH2-mediated promoter methylation [112,113].

### 3.2. BRCA1 in Histone Acetylation

Acetylated histones destabilize nucleosomes, increases chromatin accessibility for transcription factor binding, and ultimately results in increased transcriptional activity [116,117]. Histone acetylation is a reversible, dynamic event regulated by histone acetyltransferases (HAT) and histone deacetylase complex (HDAC), which adds and removes acetyl groups from histone tails, respectively. BRCA1 interacts with CBP and p300, two structurally related HATs [33]. The interactions are through both the N- and C-termini of BRCA1, and are shown to be independent of its phosphorylation status [33]. BRCA1 and p300 co-localize in the nucleus, and the transcriptional activation ability of BRCA1 is further stimulated by p300 [33].

BRCA1 interacts with HDAC1 and HDAC2, the catalytic subunits of the histone deacetylase complex, through its C-terminal BRCT domain [36]. One example of the functional outcomes of the BRCA1/HDAC interaction was demonstrated by Zheng et al [67]. Wild-type BRCA1, but not clinically validated mutants, mediates ligand-independent transcriptional repression of ERα [67]. The BRCA1-dependent ERα repression is largely restored by HDAC inhibitor trichostatin A, implicating HDAC in the process [67]. In an independent study, the interaction between BRCA1 and HDAC2 was also shown to epigenetically repress a bona fide oncomir, miR-155 [118]. BRCA1 represses miR-155 expression in human breast cancer cell lines, and treatment with HDAC inhibitors rescues miR-155 level in wild-type, but not BRCA1-deficient cells [118]. Further investigation showed that BRCA1 binds to miR-155 promoter and recruits HDAC2 to deacetylate histones H2A and H3, which in turn represses miR-155 expression [118]. R1699Q, a BRCA1 mutant carrying a mutation in its BRCT domain, loses its interaction with HDAC2 [118]. R1699Q is associated with the miR-155 promoter at a similar level as wild-type BRCA1, yet fails to recruit HDAC2 to the promoter [118]. R1699Q-expressing cells show increased acetylation of H2A and H3 at the miR-155 promoter and upregulation of miR-155 [118]. It is worth noting that the R1699Q mutant leads to moderate risk of breast cancer, while showing no substantial defects in sensitivity to DNA damaging agents, cell growth or overall genomic stability [118,119]. In addition, knockdown of oncomir miR-155 in BRCA1-deficient cells significantly inhibits in vivo tumor growth [118]. Taken together, these findings suggest a role for BRCA1 in the epigenetic control of an oncogenic microRNA through histone deacetylation [118].

### 3.3. BRCA1 in Histone Ubiquitination

The 76-amino acid protein ubiquitin can be conjugated to all subunits of the histone octamer [120]. The most common types of histone ubiquitination are the monoubiquitination of histone H2A and H2B, which comprise about 5–15% of total H2A and 1–2% of total H2B in the nucleus [120]. Histone ubiquitination plays critical roles in transcription, maintenance of chromatin structure, and DNA damage response [120]. The N-terminal RING domain of BRCA1 is responsible for its E3 ubiquitin ligase activity [121]. BRCA1, along with its heterodimeric partner BARD1, transfers ubiquitin from its interacting E2 ubiquitin-conjugating enzymes to its targets [121,122]. Although BRCA1/BARD1 ubiquitylates both H2A and H2B in vitro without any apparent preference, it has been shown that in a nucleosomal context the BRCA1/BARD1 complex specifically ubiquitylates chromatin-associated H2A at lysine 127 and 129 in vitro and in vivo [122,123,124]. The ability to distinguish nucleosome substrates from free histones resides in the BRCA1/BARD1 heterodimeric RING domains [123]. In a more recent study, BRCA1/BARD1-mediated H2A ubiquitination was shown to promote 53BP1 repositioning and DNA resection [125]. Lysine 123 of the histone variant macroH2A1 is also a BRCA1/BARD1 ubiquitination substrate in vitro and in vivo [126]. Primary human fibroblasts expressing ubiquitination-deficient macroH2A1 mutant are defective in cellular senescence, indicating that macroH2A1 ubiquitination plays an important role in replicative senescence [126]. It is worth noting that the transcriptional preinitiation complex (PIC) is also a target of BRCA1-mediated ubiquitination. Ubiquitylated PIC prevents the assembly of basal transcription factors at the promoter, and thus represses transcription initiation [127].

The role of BRCA1 in histone ubiquitination could contribute to its tumor suppressor function [128]. BRCA1 deficient cells exhibit reduced H2A ubiquitination at major and minor satellite repeats and dramatically induced normally silenced satellite transcripts [128]. A polymorphic BRCA1 variant V11A, but not ubiquitin ligase-defective mutant T37R, represses the satellite transcripts to a similar extent as wild-type BRCA1, indicating that the ubiquitination function of BRCA1 is essential for satellite DNA repression [128]. Importantly, ubiquitin-fused H2A (H2A-Ub) that mimics natural monoubiquitylated H2A restores satellite DNA silencing in BRCA1-deficient cells [128]. H2A-Ub fusion also rescues *BRCA1* deletion-induced proliferation defect and apoptosis induction, and at least partly restores impaired homologous recombination associated with loss of BRCA1. Furthermore, ectopically expressed satellite RNA partially phenocopies BRCA1 loss, including centrosome amplification, cell-cycle checkpoint defects, and γH2AX foci formation [128]. Collectively, these findings suggest that impaired H2A ubiquitination-mediated satellite DNA suppression is associated of BRCA1-related defects, providing a potential new function of BRCA1 in tumor suppression.

## 4. BRCA1 in Chromatin Reorganization

Eukaryotic chromatin is organized into euchromatin and heterochromatin regions. In general, euchromatin regions are more accessible and transcriptionally active, while heterochromatin regions are more condensed and transcriptionally silent [90]. Heterochromatic regions are enriched for repetitive DNA sequences that are normally silenced, including satellite repeats and transposable elements [90]. Histone hypoacetylation, histone H3 lysine 9 hypermethylation, DNA methylation and HP1 binding are all characteristics of heterochromatin [90]. Chromatin organization in eukaryotic cells is under tight regulation to ensure proper transcription and other chromatin-associated events. Chromatin remodeling complexes and epigenetics-modifying enzymes control nucleosome packaging and chromatin structures [129]. A number of studies have linked BRCA1 with chromatin regulation through its interaction with chromatin remodelers, epigenetic modifiers, and its action in histone ubiquitination. 

When artificially tethered to chromatin in budding yeast, the C-terminal transcriptional activation domain of BRCA1 is shown to alter local chromatin structure [130,131]. Wild-type BRCA1, but not cancer-predisposing mutants, possesses the chromatin remodeling ability [130]. BRCA1 is also found to be associated with the SWI/SNF chromatin remodeling complex through a direct interaction with BRG1, the essential ATPase subunit of the SWI/SNF complex [132]. The ability of BRCA1 to stimulate p53-dependent transcription is completely abrogated by either a *BRCA1* exon 11 deletion mutant or a dominant-negative ATPase mutate of BRG1, indicating that the p53-mediated coactivation function of BRCA1 is through SWI/SNF complex, possibly by chromatin remodeling [132]. Of note, the BRCA1-SWI/SNF complex represents the predominant BRCA1-containing complex in the HeLa nuclear extract [132]. Using a unique lac-based chromatin-tethering system, Ye et al. demonstrated that BRCA1 induces large-scale chromatin decondensation when targeted into the mammalian genome [34]. The chromatin-decondensing activity is mapped to the C-terminal domains of BRCA1 [34]. Somewhat paradoxically, cancer-predisposing mutations in BRCT domains significantly enhance the chromatin-unfolding activity of BRCA1 [34]. Taken together, these reports support a role of BRCA1 in influencing chromatin organization.

Disrupted heterochromatin silencing is reported in BRCA1-deficient mouse brains, fibroblasts, mammary glands, and human cancer cells [103,128,133]. These BRCA1-deficient cells exhibit reduced heterochromatin foci number, decreased HP1-positive foci number, and loss of transcriptional silencing of tandemly repeated DNA [103,128,133]. Zhu et al. attributes ubiquitin ligase function of BRCA1 to heterochromatin silencing [128]. In particular, depletion of BARD1, the heterodimeric E3 partner of BRCA1, alleviates suppression of satellite DNA transcription similar to the effect of BRCA1 depletion [128]. Furthermore, wild-type BRCA1, but not a pathogenic ubiquitin ligase dead mutant, represses repetitive DNA transcripts [128]. Lastly, H2A-ubiquitin fusion restores satellite DNA silencing in BRCA1-deficient cells [128]. These observations indicate that BRCA1 controls heterochromatin silencing through its ubiquitin E3 ligase-mediated histone H2A ubiquitination.

## 5. Conclusions and Future Perspectives

**Figure 3:**
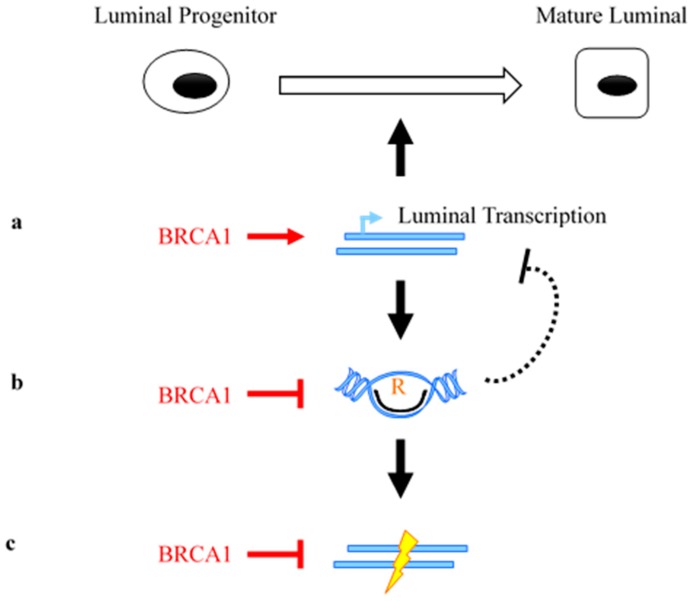
Model depicting BRCA1-dependent transcriptional regulation in tissue-specific tumor suppression. (**a**) BRCA1 is required for luminal differentiation. (**b**) BRCA1 prevents R-loop accumulation in the luminal lineage. (**c**) BRCA1 repairs double-strand breaks.

A role of BRCA1 in transcription regulation, combined with that in maintenance of genome integrity, could provide a better molecular explanation for its tissue- and lineage-specific tumor suppressor function. Here we propose a model that seeks to integrate multiple activities of BRCA1 in these molecular processes (Figure 3). First, we propose that BRCA1 promotes luminal-fate gene transcription (Figure 3a), which results in differentiation from luminal progenitors to mature luminal cells. Second, BRCA1 prevents R-loop accumulation, preferentially at the luminal-fate genes, through its functional interplay with a transcriptional pausing factor (Figure 3b) [85]. The R loop-attenuating function of BRCA1 could serve two purposes: (1) mitigating putative inhibition of luminal gene transcription by R-loops (Figure 3b) [88], and (2) reducing a potential source of DNA lesions including double strand breaks (DSB) [134]. In the event of DSB, the well-documented BRCA1 activity in HR repair provides yet another layer of protection against genomic instability during breast tissue development.

Our proposed model raises several important questions. First, is there more compelling evidence for a direct role of BRCA1 in regulation of luminal gene transcription? If such a role can be demonstrated, what is the underlying mechanism(s)? Currently there lacks convincing data for a stand-alone transcriptional activity of BRCA1 in normal breast epithelial cells. In this regard, separation-of-function BRCA1 mutants that abolish one but not all BRCA1 functions would be useful. Second important question concerns the cause for preferential accumulation of R-loops in the luminal cells of *BRCA1* mutation carriers. Could this be due to higher global transcription level in luminal cells compared to basal cells [135], or more promoter-paused Pol II in luminal versus basal cells? Of note, the recently reported role of BRCA1 in repairing estrogen-associated DNA damage at ERα-regulated transcriptional promoters could provide an alternative mechanism for luminal lineage-specific BRCA1 function [136]. Lastly, the fact that *BRCA1* mutations preferentially increase the risk of both breast and ovarian cancers begs the question of whether a common mechanism(s) is used by BRCA1 to regulate tissue-specific transcription in breast and ovaries. Future studies combining more sophisticated in vitro and in vivo model systems with clinical specimens are likely to provide more mechanistic insight into the multifactorial functions of BRCA1 in the physiologically relevant cell and tissue contexts.
